# The Use of Antioxidants in the Treatment of Migraine

**DOI:** 10.3390/antiox9020116

**Published:** 2020-01-28

**Authors:** Marta Goschorska, Izabela Gutowska, Irena Baranowska-Bosiacka, Katarzyna Barczak, Dariusz Chlubek

**Affiliations:** 1 Department of Biochemistry and Medical Chemistry, Pomeranian Medical University in Szczecin, Powst. Wlkp. 72, 70-111 Szczecin, Poland; irena.bosiacka@pum.edu.pl (I.B.-B.); dchlubek@pum.edu.pl (D.C.); 2 Department of Medical Chemistry, Pomeranian Medical University in Szczecin, Powst. Wlkp. 72, 70-111 Szczecin, Poland; gutowska@pum.edu.pl; 3 Department of Conservative Dentistry and Endodontics, Pomeranian Medical University in Szczecin, Powst. Wlkp. 72, 70-111 Szczecin, Poland; kasiabarczak@vp.pl

**Keywords:** migraine, migraine prophylaxis, antioxidants, oxidative stress, curcumin, Ginkgo biloba, ginkgolide B, coenzyme Q10, feverfew

## Abstract

Despite numerous studies concerning the pathophysiology of migraine, the exact molecular mechanism of disturbances underlying migraine is still unknown. Furthermore, oxidative stress is considered to play a significant role in migraine pathogenesis. The notion of oxidative stress in migraine patients has been discussed for several decades. Over the past few years, among the substances that could potentially be used for migraine treatment, particular attention has been paid to the so-called nutraceutics, including antioxidants. Antioxidants supplied with food prevent oxidative stress by inhibiting initiation, propagation, and the oxidative chain reaction itself. Additionally, the agents used so far in the prevention of migraine indeed show some anti-oxidative action. The antioxidants discussed in the present paper are increasingly more often used by migraine patients not only due to mild or even a lack of side effects but also because of their effectiveness (decreased frequency of migraine episodes or shortening of an episode duration). The present review provides a summary of the studies on nutraceuticals with antioxidative properties.

## 1. Characteristics of Migraine

Migraine can be described as a complex multifactorial neurovascular brain disorder characterized by impaired information processing in the brain, as well as recurrent unilateral hemi-cranial pain occurring in attacks [1]. The estimated prevalence of migraine is from 12% to 16% of the population, higher in women than in men (3:1), and shows a tendency for familial occurrence [2,3,4]. In the first decade of the 21st century, the possible lifetime incidence was estimated to be approximately 50% in women and approximately 20% in men [5]. The episodic form of migraine is characterized by a unilateral pulsating/throbbing headache typically lasting from 4 to 72 h, usually accompanied by photosensitivity and phonosensitivity [6]. Approximately 2% of the world’s population is affected by a chronic migraine, i.e., complication of a sporadic migraine [2].

Around 30% of patients with a diagnosed migraine suffers from the so-called migraine with aura [7,8]. The duration of a migraine aura is typically less than an hour. Following the aura, the patients experience migraine headache (less often, tension-like headache) [9]. Among others, clinical characteristics of the migraine aura include transient visual and sensory disturbances (uni- or bilateral) as well as motor symptoms due to recurrent brain dysfunction. Visual disturbances are reported most frequently [10]. Medicines aimed at preventing the attacks of migraine with aura are generally introduced in the case of an increased frequency of migraine attacks with aura, e.g., lamotrigine. Aura may contribute to the so-called migrainous stroke, i.e., an episode of cerebral ischemia with neurological deficits [11]. The pathophysiology of migraine aura is connected with the slow depolarization wave propagating across the cortex, known as cortical spreading depression (CSD). According to a few researchers, CSD is also implicated in the pathogenesis of headaches [12,13]. 

**Figure 1:**
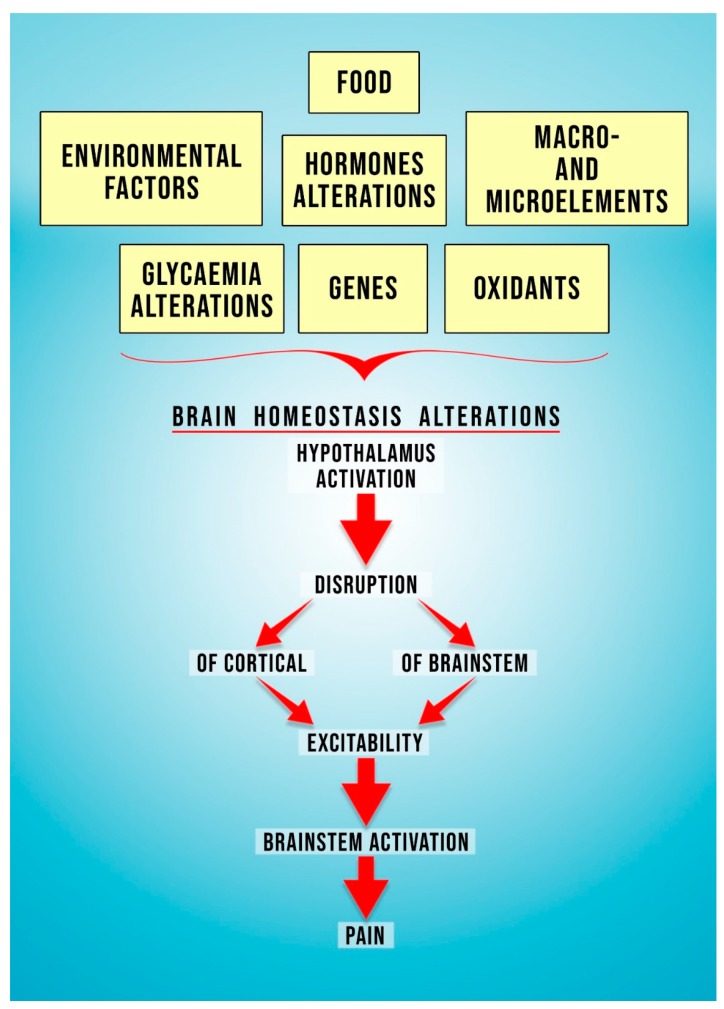
Neurobiological processes leading to migraine pain.

As indicated above, migraine is a brain disorder related to disturbances in the brain homeostasis and leading to, among others, activation of the trigeminovascular system, stimulation of cerebral vessels’ nociceptors, and further signal transmission in the brain. Thus, further signal transmission results in the stimulation of particular regions of the brain responsible for the clinical manifestations of migraine (triggering pain symptoms, experiencing pain or neurological symptoms) [14] (Figure 1). However, the mechanisms involved in the pathophysiology of migraine have not yet been clearly defined [14,15].

The occurrence of migraine is related with cortical hyper-reactivity, during which the brain is more sensitive to stimuli. Most likely, this is connected with a decreased ability to control the interaction between neurons (through inhibition) or with preactivation by the brain stem or thalamus [10]. CSD accompanying migraine with aura is presumably related to the activation of the afferent fibers of the trigeminal ganglion responsible for innervation of the meninges. The aforementioned activation may consequently result in the release of substance P, neurokinin A, or calcitonin gene-related peptide (CGRP) [16]. In turn, the release of the aforementioned cytokines results in endothelium and platelet activation and, consequently, an increase in NO synthesis and vasodilation, leading to clinical manifestation of migraine [16]. Additionally, GABA receptors as well as amino acids (glutamic and aspartic acid) are believed to be involved in the etiopathogenesis of migraine [17,18,19]. Head examination of migraine patients conducted using phosphorus magnetic resonance spectroscopy (31P-MRS) also showed an impaired brain energy and oxygen metabolism [20,21]. This was also confirmed by Gross et al., who indicated that migraine can, in some part, be related to energy deficits in the brain and the experienced pain stems from brain hypometabolism and oxidative stress triggered by the factors commonly causing headache (e.g., fasting, physical exertion, excess sleep or sleep deprivation, intense aromas, or photosensitivity) due to their direct or indirect effect on energy metabolism and mitochondrial activity [22].

The exact mechanism underlying the migraine pathogenesis is not clearly elucidated. There are many factors said to be engaged in the migraine etiopathogenesis: Genes, food, oxidants, environmental factors, macro- and microelements, metabolic disturbances (glycaemia alterations), or hormone alterations (yellow boxes at the top of the picture). All these factors influence brain homeostasis (red arrow). In consequence, hypothalamus activation occurs (red arrow). This leads to disruption and alteration of the cortical and brainstem excitability (red arrows). Consequently, brainstem activation takes place (red arrow). The above-mentioned neurobiological processes will result in migraine pain.

## 2. The Role of Oxidative Stress in the Pathophysiology of Migraine

Despite numerous studies concerning the pathophysiology of migraine, the exact molecular mechanism of disturbances underlying migraine is still unknown [1,8]. The speculated theories pay significant attention to oxidative stress [23,24], understood as disturbances in the reactive oxygen species (ROS) production–degradation balance [25]. As such, this phenomenon is involved in the etiopathogenesis of numerous diseases, such as atherosclerosis, reduced kidney function, or ischemic stroke [26,27,28]. Recently, it is also considered to be one of the factors leading to the development of neurodegenerative and neuroinflammatory diseases of the central nervous system, e.g., multiple sclerosis, Alzheimer’s disease, or Parkinson’s disease [25,29,30]. 

The notion of oxidative stress in migraine patients has been discussed for several decades. In the 1990s, Tozzi-Ciancarelli et al. [31] reported an increased concentration of substances reacting with thiobarbituric acid in patients suffering from migraine with aura during attack-free periods [31]. Several years later, Alp et al. [32] conducted a study on the indices of cell redox status in migraine patients and showed differences in the total antioxidant status (TAS), total oxidant status (TOS), and oxidative stress index (OSI). The recorded TAS values were lower, whereas TOS values were found to be markedly higher in patients with diagnosed migraine without aura as compared with the control [32]. In 2016, Geyik et al. investigated migraine patients (both with and without aura, divided into respective subgroups) not only in terms of the parameters of oxidative stress but also with respect to oxidative stress-dependent DNA damage measured with 8-hydroxy-2′-deoxyguanosine (8-OHdG) levels [33]. The obtained results showed a lack of differences in TOS, TAS, and OSI values between the study group and the control (healthy individuals without migraine). Moreover, there were no differences in TOS, TAS, and OSI values between the groups of patients suffering from migraine with and without aura [33]. However, a significantly higher 8-OHdG plasma concentration was observed in the migraine patients as compared with the control, as well as in the group of patients with migraine without aura in comparison with the group of patients with migraine with aura [33].

In 2018, in their study on patients with diagnosed migraine, Yigit et al. aimed to establish plasma lymphocytes’ damage, which is significantly affected by oxidative stress. The authors determined serum levels of urotensin receptor (UTS2R), a peptide present in the heart and blood vessels. The said peptide has strong vasoconstrictive properties, and its elevated concentration was found in patients suffering from migraine without aura [34]. Additionally, the study was supplemented with identification of the plasma MDA concentration; levels of TOS, TAS, and OSI; as well as CAT activity [34]. The results obtained in the course of this study showed significantly elevated values of TOS and OSI, increased levels of lymphocyte DNA damage, and an elevated MDA concentration in migraine patients as compared with the control. However, the levels of TAS, UTS2R, or CAT activity were found to be lower in the study group as compared with the control [34].

The study by Aytaç et al. on migraine patients with diagnosed or excluded white matter hyperintesities (WHM) showed that migraine patients with a high level of oxidative stress are characterized by an increased risk of the occurrence of white matter hyperintensities [35]. This was confirmed by the obtained results of lower CAT activity and higher plasma MDA concentration in the study group as compared with the control. Additionally, the study found that migraine patients with WHM-type lesions were characterized by lower CAT activity and elevated MDA concentration in comparison with migraine patients without WHM-type lesions [35].

The aforementioned results obtained in the course of studies on oxidative stress parameters in migraine patients seem to confirm the involvement of oxidative stress in the pathogenesis of migraine (Table 1).

## 3. Migraine and Plant Antioxidants

Migraine is a multifactorial disorder with a complex of mechanisms engaged in its pathogenesis [1,14,15]. That is why there is no single treatment that would be effective in every migraineur. For acute treatment, triptans have been recommended for several dozens of years [36]. Patients experiencing frequent attacks of migraine, which have a significant effect on their quality of life, require preventive treatment. Those who experience sporadic migraine attacks usually need rescue medication to stop the pain [37]. However, due to the side effects reported when using the standard prophylactic medication (antiepileptic drugs, calcium channel inhibitors) [38,39] or abortive therapy, the discovery of substances with a more gentle effect and better tolerance is still required, i.e., causing less side effects [40]. Given the prevalence of migraine in children, this issue is of particular relevance [41].

Over the past few years, among the substances that could potentially be used for migraine treatment, particular attention has been paid to the so-called nutraceutics [42]. This group includes, among others, vitamins (e.g., riboflavin), dietary supplements with, for example, coenzyme Q10, and alpha lipoic acid [42,43]. Antioxidants supplied with food prevent oxidative stress by inhibiting initiation, propagation, and the oxidative chain reaction itself. Other mechanisms of action of antioxidants from food are, among others, the scavenging of free radicals, molecular oxygen quenching, and acting as reductants in oxidative reactions [44]. Ongoing studies on the mechanisms of migraine pathogenesis have contributed to advances in research on possible treatment modalities. In the present state of knowledge, it is believed that the pathogenesis of migraine, in all its complexity, is determined by, among others, genetic and epigenetic factors as well as the effect of numerous environmental factors [43]. Furthermore, as mentioned above, oxidative stress is considered to play a significant role in migraine pathogenesis. The modulation of the effect of oxidative stress is possible by providing antioxidants [43]. Additionally, the agents used so far in the prevention of migraine indeed show some antioxidative action [43].

Apart from supplementation, dietary sources of natural antioxidants particularly include products of plant origin, vegetables, fruits, flowers of edible plants, and plant-derived spices [45,46]. The most common plant antioxidants are carotenoids (xantophylls and carotenes), polyphenols (phenolic acid, anthocyanins, lignans, flavonoids, and phenolic acid), and vitamins C and E [47,48,49].

## 4. Vitamin C

Water-soluble vitamin C is one of the most vital nonenzymatic antioxidants. Since the human organism lacks the enzyme necessary for vitamin C synthesis, i.e., L-glucono-gamma-lactone-oxidase, it needs to be provided with food [50]. Dietary sources of vitamin C are fruits and vegetables [51]. Ascorbic acid (L-ascorbic acid; LAA), being a weak acid, is the active form of vitamin C. This is an unstable organic compound, which is easily decomposed due to temperature, light, the activity of heavy metals, and pH [50]. Ascorbic acid is involved in, among others, detoxification processes in the human body and neuronal metabolism [52]. The effect of LAA on the redox balance in the human body is twofold: It acts as a reducing agent and is an enzyme cofactor [53]. At the same time, due to its reducing properties, LAA can reduce metals, such as iron or copper, thus increasing their oxidizing properties [53]. 

Epidemiological studies conducted on patients suffering from migraine, inflammatory bowel disease, and asthma showed an increased risk of developing the so-called complex regional pain syndrome (CRPS) in the limbs owing to the destruction of nerve fibers [54]. Following wrist injury, administering vitamin C as a daily dose from 200 to 1500 µg in a period of up to 50 days reduced the risk of CRPS and consequently was approved as a prophylactic treatment of CRPS [54]. The results obtained in the aforementioned study prompted scientists to stipulate that the administration of vitamin C may also modulate the effects of neuroinflammation and ROS activity in the course of migraine [54]. The analysis of the effectiveness of vitamin C in shingles pain by Kim et al. proved the efficiency of intravenously administered vitamin C (at a dose of 5 g/day on the first and the third day of experiencing pain) in the prophylactic treatment of episodes of post-shingles neuralgia. However, vitamin C administration had no effect on relieving pain episodes due to *Herpes zoster* infection [55]. 

In 2006, Chayasirisobhon examined the influence of an antioxidant combination product (10 capsules a day) in patients suffering from migraine (with and without aura). In this uncontrolled open-label study, patients received capsules containing 120 mg of pink bark extract, 60 mg of vitamin C, and 30 IU of vitamin E (in each capsule). In patients who completed the treatment period, improvements, including both reduced headache frequency and headache severity, were observed [56]. Chayasirisobhon also conducted an open-label study to examine the effect of vitamin C (150 mg) and *Pinus radiata* bark extract on migraine symptoms. The patients were administered vitamin C and *Pinus* bark in the aforementioned doses for 3 months. After treatment, the patients demonstrated significant improvements: Headache frequency and headache severity were reduced [57] (Table 2).

## 5. Curcumin

Curcumin is a yellow dye first isolated from turmeric (*Curcuma longa*) in the 19th century by Vogel et Pelletier [58]. Curcumin is the main curcuminoid obtained from turmeric rhizome and is one of the best-known plant polyphenols [59]. It is estimated that the total content of curcumin in turmeric rhizome is approximately 50% [60]. According to numerous sources, from isolated turmeric curcuminoids, curcumin constitutes from 60% to 85%, followed by demethoxycurcumin (15–27%) and bisdemethoxycurcumin (5–15%) [60,61]. Curcumin is widely used as a seasoning as well as in the dyeing industry [60]. Numerous beneficial properties are attributed to curcumin, such as antioxidative, antiatherosclerotic, antimicrobial, immunomodulatory, and antiageing properties [58,60,62,63]. Consequently, curcumin has become a research topic in identifying its possible use in the treatment of numerous conditions [64], for example, the prevention of diseases of the brain; spinal cord, cranial, and peripheral nerves disorders, nerve roots disorders; as well as dysfunctions of the autonomic nervous system, diseases of the neuromuscular junction, and myopathies [64]. The mechanisms of the beneficial activity of curcumin on the nervous system include limiting the production of ROS and reactive nitrogen species (RNS), and counteracting the decreased activity (SOD) and level (GSH) of antioxidants [65]. Furthermore, studies conducted on the nervous system revealed its anti-inflammatory properties owing to the inhibition of cyclooxygenase (COX-s), Il-1 I IL-6 expression, as well as antiapoptic properties [65].

Recently, the use of curcumin in migraine treatment has been increasingly considered by scientists. Bulboacă et al. conducted a study on migraine treatment in which they compared the effect of sumatriptan (ST) administered alone and ST used together with curcumin. The study was conducted using a rat model of migraine induced by nitro-glycerine. Curcumin was administered intravenously in the form of a) alcoholic solution (diluted in saline), and b) liposomes due to reduced gastrointestinal absorption of curcumin [66]. The authors reported the antioxidative effect of curcumin (decrease in MDA concentration, reduced production of RNS, reduction of TOS, and an increase in TAS) for each dose and form applied (solution/liposomes). However, greater antioxidative properties of curcumin were observed when it was administered in the form of liposomes. The authors attribute the obtained results to curcumin’s ability to scavenge hydroxyl or peroxyl radicals, direct interaction with peroxide radical anion, and inhibition of the activity of κB nuclear transcription factors (NF κBs), which are involved in the transcription of proinflammatory factors [66]. The same scientists also conducted another study using a rat model of migraine (also induced by nitro-glycerine) to compare the effect of naproxen and curcumin solution on the parameters of oxidative stress and pain sensations [67]. Administration of liposomal curcumin resulted in a decreased MDA concentration, reduced nitrogen oxide (NO) synthesis, reduced TOS values, and reduction of nociception. When curcumin and naproxen were administered together, the antioxidative mechanisms were found to be improved (thiol increase and higher total antioxidant capacity, TAC) in comparison with naproxen used alone. However, the authors indicated that a greater antioxidative effect was observed in subjects who were administered curcumin in the form of liposomes [67]. Yet another study confirmed the positive antioxidative and analgesic effect of curcumin as compared with treatment using propranolol and indomethacin [68] (Table 2).

Studies on the possible positive effects of curcumin in migraine treatment were also conducted on people. One of the considered mechanisms of migraine pathogenesis concerns the involvement of tumor necrosis factor α (TNF-α), which results in the occurrence of migraine symptoms through initiation of neuronal hyper-excitability, stimulation of nociceptors, and prostanoid production which, in turn, leads to initiation of neuroinflammation [69]. Abdolahi et al. conducted a study on a group of patients with diagnosed sporadic/episodic migraine, with the aim of identifying the synergistic effect of curcumin and ω-3 acids on gene expression for TNF-α. Patients receiving both substances showed a reduction of mRNA levels for TNF-α in plasma (which reflects a decrease in TNF- α expression). This was not found in the case of patients receiving only one of the analyzed substances [69] (Table 2).

The study on the antioxidative effects of nanocurcumin used jointly with coenzyme Q10 (another popular preventive anti-inflammatory agent) was conducted on patients with sporadic migraine. The results of this study show that joint administration of curcumin and coenzyme Q10 has a positive effect on decreasing the frequency of migraine attacks, their duration, and severity of symptoms. Additionally, the patients did not report side effects with respect to the antioxidants used in the study [70] (Table 2).

## 6. Coenzyme Q10

Coenzyme Q10, also known as ubiquinone, is an endogenously produced lipid compound, which contains 10 isoprenoid units in its molecule [71], and is lipid soluble [72]. It shows antioxidative properties and provides protection to cells against excessive ROS production, which prevents excessive oxidation of nucleic acids or lipid membrane peroxidation. Apart from its antioxidative properties, coenzyme Q10 also shows some anti-inflammatory properties [72], is involved in pyrimidine synthesis (as cofactor), and, in turn, in DNA replication and RNA repair processes. Additionally, it is a modulatory factor of the physicochemical properties of cell membranes and gene expression [73,74]. Coenzyme Q10 is a relevant element in the respiratory chain, where it acts as an electron carrier [74]. Due to its potential positive effects, it is considered as a dietary supplement in numerous diseases of the nervous system, for example, in neurodegenerative diseases (Parkinson’s diseases, amyotrophic lateral sclerosis (ALS), Friedreich’s ataxia) and in multiple sclerosis (MS) [71,75].

Coenzyme Q10 is a factor that has an effect on sustaining mitochondrial metabolism and, given one of the theories on migraine pathogenesis stipulating the disturbance of mitochondrial metabolism, it is used and analyzed in terms of possible applications in migraine treatment [76]. Following administration of coenzyme Q10 to patients with rheumatoid fibromyalgia (treated with pregabalin), the concentration of the reduced form of glutathione and SOD activity was increased, pain sensation and anxiety symptoms were relieved, and the parameters of mitochondrial oxidative stress and inflammation were lower [77].

According to experts’ recommendations (e.g., American Academy of Neurology), the first-line treatment medications in migraine prevention are: β-blockers (propranolol), antiepileptic drugs (topiramate, valproic acid), and antidepressants (amitriptyline). Nutraceuticals, such as coenzyme Q10, offer an alternative to the aforementioned first-line treatment drugs, mostly owing to appreciably less or even a lack of side effects [78]. Indeed, coenzyme Q10 is listed as one of the most commonly used treatments in migraine prevention [20,79]. Recently, increasingly more attention is paid to the fact that coenzyme Q10 administered to migraine patients results in a decrease of calcitonin gene-related peptide (CGRP), which determines the CGRP level as the goal of prophylactic treatment for migraine [80] (Table 2).

In the first decade of the 21st century, Hershey et al. conducted studies on pediatric and adolescent migraineurs complaining of frequent headaches and showing low plasma coenzyme Q10 levels [81]. The authors demonstrated that supplementation with coenzyme Q10 at a dose of 1–3 mg/kg body weight (gel capsule) resulted in an increase of the plasma coenzyme Q10 concentration and reduced the frequency of headache episodes. Additionally, it was found that headache disability status measured with the Migraine Disability Assessment Scale in pediatric and adolescent patients (PedMIDAS) was improved. The authors suggested that coenzyme Q10 deficits can be a cause of migraine headaches in children and adolescents, thus stressing the importance of determining coenzyme Q10 deficiency levels in those patients and respective supplementation [81] (Table 2). Additionally, Zeng et al., on the grounds of their studies on migraine patients, suggested a positive effect of coenzyme Q10 supplementation in terms of reducing the duration of migraine attacks as well as their number in a given time [82] (Table 2).

The relationship between supplementation with coenzyme Q10 and the occurrence of migraine was also studied in children and adolescents by Slater et al. [83]. The patients were given a daily dose of 100 mg of coenzyme Q10, for a period of 4 months. The results did not show statistically significant differences between the groups in terms of the primary outcome. However, the study group of migraine patients showed a decreasing trend in terms of the frequency of headache episodes as compared with the control. Moreover, in the first four weeks of supplementation with coenzyme Q10, a decreased severity of headache in patients with episodic migraine was observed. However, when discussing the results of the aforementioned study, it must be emphasized that the study was completed by merely 52% of the patients (62 out of a total 100), which could have affected the results of the study [83] (Table 2).

Guilbot et al. analyzed the effects of a trivalent supplementation with Antemig^®^ specimen (coenzyme Q10, magnesium and feverfew) in adults with diagnosed migraine (according to the classification by the International Headache Society) [81]. After three months of supplementation, the study showed a lower number of days with migraine per month as well as reduced signs of anxiety and depressive symptoms. Three-month-long supplementation also resulted in a significant reduction in symptoms, such as photosensitivity and nausea, and an improved quality of life (assessed with the Qualité de Vie et Migraine (QVM) questionnaire) [84] (Table 2).

In turn, apart from the supplementation of coenzyme Q10, Gaul et al. analyzed the effects of supplementation with riboflavin and magnesium (dietary supplement sold under the brand name of Migravent in Germany and Dolovent in the USA) on the frequency of migraine episodes. The authors demonstrated that daily supplementation with the dose of 150 mg of coenzyme Q10, 400 mg of riboflavin, and 600 mg of magnesium together with other multivitamins, did not produce a sufficient reduction in the number of days with migraine. However, after three months of supplementation, reduced severity of migraine was observed. No significant side effects were reported, apart from abdominal discomfort and diarrhea (which was attributed to a high daily dose of magnesium) [78] (Table 2).

A notable effect of supplementation in migraine prophylaxis in adults was also observed by Shoeibi et al. [85]. The open-label, parallel-add-on, and match-controlled study demonstrated that administration of coenzyme Q10 at a dose of 100 mg/day (together with previously taken preventive drugs) resulted in a reduction of the frequency of migraine episodes and, simultaneously, shortening of the duration of a single episode. Additionally, in the group of patients taking coenzyme Q10 supplements, there was a reduction of symptoms, such as nausea, photosensitivity, and phonosensitivity. Given the obtained results, it was suggested that migraine prevention based on the use of coenzyme Q10 could be potentially beneficial particularly to patients with rare yet severe episodes of migraine [85] (Table 2).

## 7. Ginkgolide B

**Figure 2:**
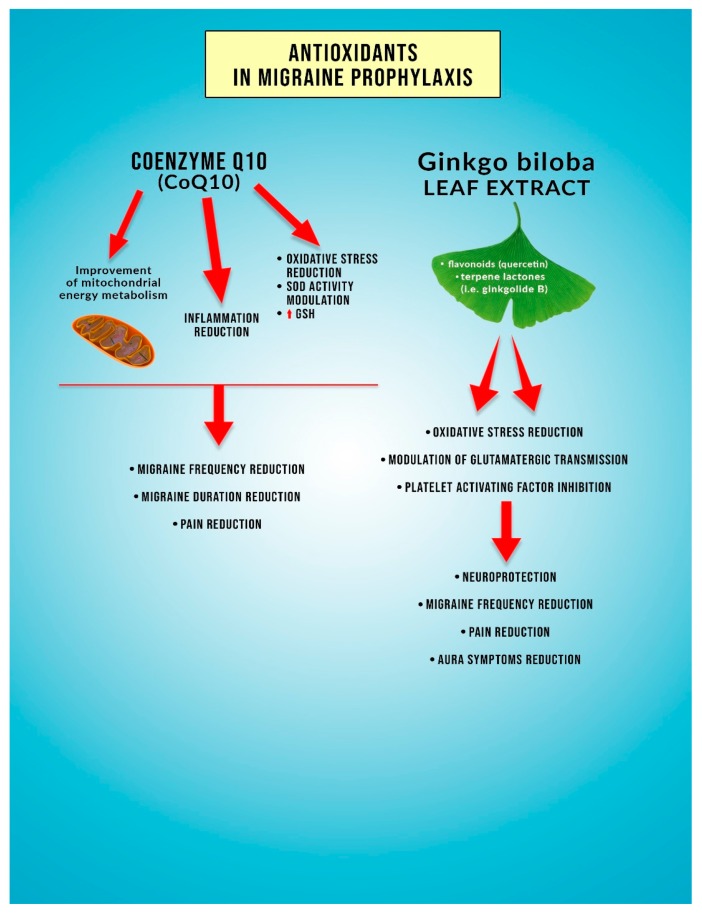
Influence of coenzyme Q10 and *Ginkgo biloba* leaf extract antioxidant on red-ox status parameters in patients with migraine.

Ginkgolide B is an herbal component of the extract obtained from *Ginkgo biloba* (GB) leaves. It is one of the most popular and commonly used products of plant origin. Its regular and long-term administration is a component of primary and secondary prevention of numerous conditions, such as depression, anxiety, headache, and memory deficits [86]. The literature on the subject stresses the neuroprotective properties of the extract from *Ginkgo biloba* leaves; however, the exact mechanism of its neuroprotective action requires further investigation [87,88]. It is proven that due to its antioxidative and anti-inflammatory activity, an extract of GB leaves contributed to reduced hippocampal neuronal death, a consequence of transient global ischemia (TGA) [89]. The essential biologically active substances of the extract of GB leaves are flavonoids and terpene lactones [90]. Some authors attribute the antioxidative action to quercetin rather than ginkgolide B [91]. However, ginkgolide B is the most often listed nutraceutical agent used in migraine treatment [92]. Although its main mode of action in migraine is believed to be modulation of brain glutamatergic transmission and platelet-activating factor (PAF) receptor antagonism [89], the literature on the subject also reports its antioxidative properties [91,93,94] (Figure 2). This seems critical given the changes in the redox status in the course of the migraine. Next to ginkgolide A, C, and terpenes, ginkgolide B is one of the main active ingredients of GB leave extract showing beneficial effects on memory [87].

D’Andrea et al. [95] conducted a six-month-long open-label multicenter study, which demonstrated the efficiency of ginkgolide B in the treatment of migraine with aura in patients who experienced migraine episodes at least once a month. The patients included in the study were healthy individuals, without any comorbidities, particularly without cerebral focal activation. For the following four months (the study period was divided into two two-month-long phases), the patients took Migrasoll^®^ preparation twice a day (60 mg of *Ginkgo biloba* terpenes phytosome, 11 mg of CoQ10, 8.7 mg of vitamin B2). The study group patients showed a significant reduction in the frequency of migraine episodes, as 42.2% of patients reported a lack of migraine episodes following the termination of the study, and in 5 patients there was no reaction to the applied prophylactic treatment with Migrasoll^®^. Moreover, introducing supplementation resulted in a shortening of the duration of aura throughout the entire study period [95] (Table 2). A similar study with the use of Migrasoll^®^ was also conducted by Allais et al. [11] (Table 2).

During the first migraine episode, patients included in the aforementioned study were instructed to record the symptoms of aura and severity of pain. During the second (following) migraine episode, the patients were advised to take two capsules of Migrasoll^®^ orally at the first symptoms of aura. The use of analgesics by the patients was continued. The results showed that taking Migrasoll^®^ resulted in a significant shortening of aura duration (in minutes). Furthermore, in four migraine patients with a standard course of aura, the pain phase was resolved [11] (Table 2).

Coenzyme Q10 (left side of the graph) modulates energy metabolism within mitochondrion, thus leading to the improvement of the energy metabolism of this organelle (first from the left red arrow). Impairment of the energy metabolism of the mitochondrion is said to be involved in migraine pathogenesis. Coenzyme Q10 possesses anti-inflammatory as well antioxidative properties (second from the left red arrow). Among its antioxidative properties, the following are described, i.e., modulation of superoxide dismutase activity (leading to improvement of the antioxidative defense of the cell), reduction of the oxidative stress (OS), or ability to increase the concentration of the reduced form of the glutathione (GSH) (which serves as the antioxidant) (third red arrow). The biological mechanism described above leads to a reduction of migraine attacks’ frequency, to the shortening of migraine attacks’ duration, and to the alleviation of migraine pain (red arrows on the left side of the graph).

On the right side of the graph, certain properties of antioxidants present in the *Ginkgo biloba* leaf extract are mentioned. *Ginkgo biloba* leaf extract mainly contains flavonoids (i.e., quercetin) and terpene lactones (i.e., ginkgolide B) (red arrow on the right side of the graph). Quercetin and ginkgolide B are said to have a particular participation in migraine symptoms’ reduction. *Ginkgo biloba* leaf extract ingredients cause a reduction of the oxidative stress, inhibit platelet-activating factor (PAF), and influence glutamatergic transmission (red arrows on the right side of the graph). All the biological mechanisms result in a neuroprotection increase and lead, as a consequence, to a reduction of migraine headaches’ frequency, migraine pain reduction, and alleviation of the aura symptoms (red arrow on the right side of the graph).

## 8. Feverfew (Tanacetum parthenium)

In terms of migraine prevention, feverfew (*Tanacetum parthenium*) is a well-recognized plant [93,96]. It has been used in the treatment of numerous conditions since antiquity, not only to relieve symptoms of migraine but also to alleviate pain of another origin, inflammation, nausea, and vomiting [97]. Since the 1970s, the possible application of feverfew in headache treatment has been investigated [96]. *Tanacetum parthenium* L. (LNP-23 TP) belongs to the Asteraceae family and is widely distributed in South America. It is characterized by a high potential to inhibit aldose reductase activity and shows essential antioxidative properties [98]. The leaves of feverfew (*Tanacetum parthenium*) contain sesquiterpene lactones, out of which parthenolide is listed as the main biologically active ingredient [99]. Feverfew also contains other biologically active ingredients, such as flavonoids (luteolin, apigenin) and aromatic compounds (e.g., camphor) [96]. Wu et al. demonstrated the potent antioxidative properties of feverfew, which, according to the authors, result from the biological activity of luteolin and parthenolide, as well as the activity of other unidentified substances, the presence of which was found by high performance liquid chromatography (HPLC) analysis [96].

Due to the reported drug intolerance and side effects, feverfew is not recommended as the first-line treatment drug in migraine prevention [100]. The most commonly reported side effects of feverfew are oral ulcers, gastrointestinal irritation, allergic reactions, and rebound headache [99]. Owing to the lack of information regarding the safety of use of feverfew in pregnant and breastfeeding women, it is advised that such patients refrain from using it [99].

Pfaffenrath et al. conducted a double-blind multicenter placebo-controlled study to determine the effectiveness of a stable extract (MIG-99) of feverfew (*Tanacetum parthenium*) in decreasing the frequency of migraine episodes [101]. The study was aimed at determining the efficiency and safety of use of three MIG-99 doses, 2.08, 6.25, and 18.75 mg versus placebo, in a group of 147 patients with diagnosed migraine (with and without aura). The preparation proved to be effective only in a small number of patients who experienced at least 4 migraine episodes in a period of 28 days prior to the study. The most notable effect was recorded for the 6.25-mg dose of MIG-99. In 35% of the patients, at least one side effect was observed. The authors identified the lack of an overall significant effect of MIG-99 in prophylactic migraine treatment [101] (Table 2). Three years later, the same authors published results on the effectiveness and safety of MIG-99 preparation applied at a dose of 6.25 mg. In the group taking the preparation, there was a decrease in the frequency of migraine episodes by 1.9 per month (in the placebo group by 1.3 episode/month). Side effects were observed in more than 10% of the patients taking MIG-99. Given the overall results of the study, the authors determined that MIG-99 is effective in prophylactic migraine treatment [102] (Table 2).

*Tanacetum parthenium,* along with magnesium and 5-hydroxytryptophan, is an ingredient of Aurastop preparation used for relieving migraine aura. Volta et al. stipulated the possibility of a very early intervention and blocking the aura at this stage. The inhibition of migraine aura could have positive effects not only on the aura itself but also by reducing headache [103]. However, there are results that suggest otherwise. The randomized double-blind placebo-controlled trials by Pittler and Ernst demonstrated a lack of significant efficacy of feverfew in migraine treatment [104]. On the grounds of the results obtained in the randomized double-blind trials, the authors did not confirm the effectiveness of feverfew in migraine treatment as compared with placebo [104].

**Table 2:** Antioxidants in migraine treatment.

Antioxidants in Migraine Treatment			
Agent	Mechanism of Action	Authors of the Study	Results Obtained
Vitamin C	Antioxidant ROS scavenger	Chayasirisobhon [56] Chayasirisobhon [57]	Improvement in MIDAS score, number of headaches days, and headache severity reduction
Curcumin	Antioxidant Anti-inflammatory properties Anti-apoptotic properties	Bulboacă et al. [66] Bulboacă et al. [67] Bulboacă et al. [68]	Decrease in MDA concentration Decrease in RNS synthesis Decrease in TOS Increase in TAS Reduction of nociception Analgesic effect
		Parohan et al. [70]	Decrease in migraine attacks: Frequency Duration Severity
Coenzyme Q10	Antioxidant Anti-inflammatory properties Mitochondrial energy metabolism maintenance	Dahri et al. [80]	Decrease in CGRP level
		Hershey et al. [81]	Increase in CoQ10 plasma concentration Improvement in PedMIDAS score Reduction of headaches episodes frequency
		Zeng et al. [82]	Decrease in migraine attacks: Frequency Duration
		Slater et al. [83]	Decreased severity of headache in the four first weeks Decreasing trend in terms of the frequency of headache episodes
		Guilbot et al. [84]	Reduced number of days with migraine per month Reduced signs of anxiety and depressive symptoms Reduced photosensitivity and nausea Life quality improvement
		Gaul et al. [78]	Reduced severity of migraine
		Shoeibi [85]	Reduction of the migraine episodes frequency Reduction of the single episode duration
Ginkgolide B	Glutamatergic Transmission modulator Suggested as antioxidant	D’Andrea et al. [95]	Reduction of the migraine episodes frequency
		Allais et al. [11]	Significant shortening of aura duration
Feverfew	Antioxidant Anti-inflammation properties	Pfaffenrath et al. [101]	Lack of overall significant effect
		Diener et al. [102]	Decrease in the frequency of the migraine episodes

## 9. Conclusions

Migraine is a complex and multifactorial brain disorder, with unclear mechanisms underlying its pathogenesis. The complexity of migraine pathogenesis makes treatment (as well acute treatment as a preventative treatment) different in particular patients. Oxidative stress, understood as disturbances in the ROS production–degradation balance, is suggested to be one of the mechanisms engaged in the migraine etiopathogenesis. The involvement of oxidative stress in the migraine pathophysiology has been discussed for decades. The aforementioned studies in this review on oxidative stress parameters in migraine patients confirm the involvement of oxidative stress in the pathogenesis of migraine [31,32,33,34,35].

The currently available migraine treatments (both prophylactic as well as treatment aimed to relieve the symptoms) are based on the use of medication causing side effects, which is often a contraindication to undergoing therapy or causes its discontinuation. Therefore, with respect to migraine treatment (as well with as without aura), increasingly more attention is paid to nutraceuticals [105], particularly those with antioxidative properties. The nutraceuticals discussed in the present paper have antioxidative properties and are increasingly used by migraine patients not only due to mild or even a lack of side effects but also because of their effectiveness (decreased frequency of migraine episodes or shortening of an episode duration). Despite numerous studies conducted so far, it is recognized that the pathogenesis of migraine has not yet been clearly determined and still needs further investigation. However, as numerous studies have shown, the positive effect on nutraceuticals having antioxidative properties seems to confirm that oxidative stress plays a significant role as an etiopathogenetic mechanism of migraine.

The present review provided a summary of the studies on nutraceuticals with antioxidative properties. The results presented therein seem to indicate the possible use of nutraceuticals with antioxidative properties as an alternative to conventionally used medication in migraine treatment.

## Figures and Tables

**Table 1:** Stress and the pathophysiology of migraine.

No.	Authors	Number of Patients	Migraine	Results
1.	Tozzi-Ciancarelli et al. [31]	23	With aura	Increased concentration of substances reacting with thiobarbituric acid during attack-free periods
2.	Alp et al. [32]	75	Without aura	Decrease in total antioxidant status (TAS) Increase in total oxidant status (TOS) Increase in oxidative stress index (OSI) During attack-free period
3.	Geyik et al. [33]	50	With- and without aura	Lack of differences in TOS, TAS, and OSI values between the migraineurs and the control group No differences in TOS, TAS, and OSI values between the groups of migraineurs (with and without aura) Higher 8-OHdG plasma concentration in the migraine patients Higher 8-OHdG plasma concentration in patients with migraine without aura in comparison with the group of patients with migraine with aura
4.	Yigit et al. [34]	40	Without aura	Increase in lymphocyte DNA-damage Elevated values of TOS and OSI in migraineurs Elevated MDA concentration in plasma of migraineurs Decreased concentration of UTS2R in plasma of migraineurs Lower TAS level and decrease in CAT activity in migraineurs
5.	Aytaç et al. [35]	32 (18 with white matter hyperintensities and 14 without white matter hyperintensities) –WHM)	migraine with or without WHM	Decreased CAT activity and increased plasma MDA concentration in migraineurs Decreased CAT activity in migraine patients with WHM-type lesions in comparison with migraine patients without WHM-type lesions Elevated MDA concentration in migraine patients with WHM-type lesions in comparison with migraine patients without WHM-type lesions

## Abbreviations

CAT	catalase
CGRP	calcitonin-gene related peptide
CSD	cortical spreading depression
CoQ10	coenzyme Q10
COX	cyclooxygenase
CRPS	complex regional pain syndrome
GB	Ginkgo biloba
GSH	glutathione
HPLC	high performance liquid chromatography
LAA	L-ascorbic-acid
MDA	malondialdehyde
MIDAS	Migraine Disability Assessment Scale
NF κB	nuclear transcription factor κB
OSI	oxidative stress index
PAF	platelet activating factor
PedMIDAS	Migraine Disability Assessment Scale in pediatric and adolescent patients
QVM	Qualité de Vie et Migraine questionnaire
RNS	reactive nitrogen species
ROS	reactive oxygen species
SOD	superoxide dismutase
ST	sumatriptan
TAC	total antioxidant capacity
TAS	total antioxidant status
TOS	total oxidant status
TGA	transient global ischemia
